# sEMG-based gesture recognition using multi-stream adaptive CNNs with integrated residual modules

**DOI:** 10.3389/fbioe.2025.1487020

**Published:** 2025-04-29

**Authors:** Yutong Xia, Dawei Qiu, Cheng Zhang, Jing Liu

**Affiliations:** Shandong University of Traditional Chinese Medicine, Jinan, Shandong, China

**Keywords:** sEMG, gesture recognition, multi-stream convolutional neural network, residual modules, adaptive convolutional neural networks

## Abstract

**Introduction:**

Convolutional neural networks are widely used in gesture recognition research, which employs surface electromyography. However, when processing surface electromyography data, current deep learning models still face challenges, such as insufficient effective feature extraction, poor performance in multi-gesture recognition, and low accuracy in recognizing sparse surface electromyography.

**Methods:**

To address these issues, this study proposed a multi-stream adaptive convolutional neural networks with residual modules (MSACNN-RM) for surface electromyography gesture recognition, which integrates multiple streams of convolutional neural networks, adaptive convolutional neural networks, and residual modules to enhance the model’s feature extraction and learning capabilities. This improves the model’s ability to extract and understand complex data patterns.

**Results:**

The experimental results demonstrated that the model achieved recognition accuracies of 98.24%, 93.52%, and 92.27% respectively on the Ninapro DB1, Ninapro DB2, and Ninapro DB4 datasets. Compared with other deep learning models, MSACNN-RM achieves higher accuracy compared to existing models.

**Discussion:**

The proposed model explores features of sparse sEMG signals by leveraging multi-stream convolution, the combination of adaptive convolution modules and ResNet blocks enhances the model’s ability of extracting crucial gesture features. In the future, in order to deal with differences in sEMG signals caused by variations among individuals, a universal multi-gesture recognition algorithm should be developed. Meanwhile, the model should focus on optimizing and streamlining the network to reduce computational load.

## 1 Introduction

Surface electromyography (sEMG) records bioelectrical signals and captures electrical activity generated by motor units (including muscle fibers and neurons) during muscle movement. Skin surface electrodes are placed on the muscles to record electrical activity, which eliminates electrodes being inserted into the muscle tissue. This method provides both temporal- and frequency-domain information regarding electrical activity in the muscle and creates dynamic images of contraction and relaxation.

sEMG has extensive applications in various fields, such as human-computer interaction (HCI) (Tian et al., 2022; Hocaoglu and Patoglu, 2022), speech recognition (Li et al., 2023d), rehabilitation medicine (Copaci et al., 2022; Zhong et al., 2021), and exercise physiology (Moniri et al., 2021; Chang et al., 2023). In the HCI domain, gesture recognition based on sEMG is a popular research topic. The technology allows freedom from the constraints of traditional input devices, thereby enabling more intuitive and natural interactions. The system detects muscle activity to perceive user gestures, thereby facilitating control over external devices. In the field of speech recognition, sEMG technology can be utilized to monitor the electrical activity of throat muscles, analyzing the vibration of vocal cords and the movements of throat muscles during speech. Furthermore, sEMG can record and analyze the activity of the lip muscles, as lip movements directly affect speech production. This highlights the significant potential of sEMG in speech recognition research and development in rehabilitation medicine, where sEMG is employed to evaluate patients’ muscle function and activity levels. By monitoring muscles’ electrical activity, rehabilitation professionals can understand the patient’s muscle contraction patterns, coordination, and strength levels, thereby formulating more precise rehabilitation therapies. In exercise physiology, sEMG is utilized to monitor muscle fatigue as it develops during physical activity. By observing changes in the muscles’ electrical activity, researchers can assess the fatigue levels of athletes at different exercise intensities and durations.

As machine-learning methods have advanced, significant progress has been made in the field of sEMG gesture recognition. Advanced machine-learning algorithms enable more accurate gesture recognition by the system. Support vector machines (SVM) (Cai et al., 2019), decision trees (Song et al., 2019), the K-nearest neighbors algorithm (Zheng et al., 2022), multilayer perceptron (Luo et al., 2017), and the random forest algorithm (Wang et al., 2022a) have been applied to the prediction and estimation of sEMG motion signals. These developments have driven the widespread application of sEMG gesture-recognition technology in areas such as virtual reality, smart homes, and medical treatment.

More recently, in machine learning, deep learning technology has gained popularity among researchers owing to its excellence in automatically learning features and strong data-fitting capabilities. Deep learning models also exhibit robust adaptability and can accommodate individual diversity, muscle activity patterns, and environmental conditions, demonstrating excellent generalization capabilities. These advantages have earned deep learning high praise in sEMG recognition tasks. Techniques such as convolutional neural networks (CNNs) (Vijayvargiya et al., 2021; Li et al., 2023c), recurrent neural networks (RNNs) (Hu et al., 2018), and long short-term memory (LSTM) networks (Bai et al., 2021) have been widely applied to the motion prediction and estimation of sEMG signals, yielding favorable results.

Previous research has suggested the vast potential of sEMG signals in the HCI domain. However, owing to the inherent differences among subjects in sEMG data collection (Castellini et al., 2009) and its susceptibility to interference (Xiao, 2022), factors such as varying muscle strength among individuals (Liu et al., 2022) and different body fat percentages (Lanza et al., 2020) can affect the expression of sEMG data. Moreover, the signals collected by sEMG electrodes are likely to be influenced by electrode displacement (Wang et al., 2022c) and perspiration (Abdoli-Eramaki et al., 2012). To address these challenges, researchers have begun utilizing semi-dry electrodes and flexible electrodes. Semi-dry electrodes reduce dependence on conductive gels by combining the advantages of dry and wet electrodes while maintaining excellent signal transmission performance, thereby exhibiting high comfort and reliability in practical applications (Li et al., 2021a). Additionally, flexible electrodes are capable of conforming better to the skin surface, minimizing electrode displacement, and enhancing signal stability, thereby improving the accuracy and reliability of data acquisition (Li et al., 2023a; Li et al., 2024). Although the use of semi-dry and flexible electrodes can improve the stability and reliability of signal acquisition, reducing interference and more accurately extracting motion features from sEMG signals remains a major challenge in current gesture recognition technology.

To address these challenges, researchers have begun exploring deep learning methods to extract deeper information from sEMG signals, overcoming the limitations of traditional methods in capturing complex spatial and temporal patterns. Hu (Hu et al., 2018) proposed a hybrid CNN-RNN architecture based on an attention mechanism for sEMG-based gesture recognition. This approach leverages Convolutional Neural Networks to extract spatial features from sEMG signals and combines them with Recurrent Neural Networks (RNNs) to capture temporal sequence characteristics. Additionally, the study introduced a novel sEMG image representation method, enabling the model to learn correlations between sparse multi-channel signals. This method achieved accuracies of 87.0% and 82.2% on the Ninapro DB1 and DB2 datasets, respectively, significantly outperforming traditional methods and demonstrating an effective capability to extract complex gesture signal patterns. Although this performance is achievable under controlled laboratory conditions, practical applications, particularly in the daily use of prosthetics, still face challenges. To further enhance the performance of dynamic hand movement recognition, Yang (Yang et al., 2021) proposed a Multi-Stream Residual Long Short-Term Memory network (MResLSTM) for dynamic hand movement recognition. This architecture combines residual models and Convolutional Long Short-Term Memory networks to extract spatiotemporal features from both global and deep aspects, and preserves necessary information through feature fusion. It achieved an accuracy of 89.65% on the Ninapro DB1 dataset. It is well known that deep feature signals are critical for gesture classification. Although existing studies have integrated multi-stream convolutional neural networks and residual networks, there remains a research gap in further incorporating adaptive convolutional neural networks to enhance the model’s dynamic characteristics and robustness.

To address these issues, this study adopted a multi-stream neural network as a backbone that integrates adaptive CNNs and residual networks (ResNets). A novel network model was constructed for sEMG-based gesture recognition. The experimental results show that the network model achieved higher recognition accuracy on the Ninapro DB1, Ninapro DB2, and Ninapro DB4 datasets, demonstrating better performance compared to other models.

The contributions of this study are as follows:

1. Building on a multi-stream CNN, we addressed individual differences and susceptibility to interference in sEMG signals by introducing an adaptive CNN. This network can flexibly adjust the number and size of convolutional kernels based on the features of sEMG images and better adapt to various gesture movements. This enhances the flexibility and adaptability of gesture recognition, enabling it to handle diverse gestures.

2. To extract deep-level sEMG features, we introduced residual modules. The advantage of residual modules lies in their ability to improve the training of deep networks. The incorporation of residual modules facilitated the extraction of crucial features from sEMG signals, thereby supporting the model’s ability to capture variations in gestures. The integration of this deep-learning architecture enhances the model’s modeling and recognition capabilities for complex gestures.

3. By leveraging residual networks, multi-stream CNNs, and adaptive CNNs, we formulated the MSACNN-RM framework, resulting in a significant improvement in the accuracy of sEMG-based gesture recognition tasks. This approach effectively utilizes the deep learning capabilities of residual networks, the information fusion properties of multi-stream CNNs, and the dynamic feature learning abilities of adaptive CNNs. By amalgamating different types of neural networks, we enhanced the resolution of the sEMG signal analysis, contributing to more accurate and reliable gesture recognition in practical applications.

4. The proposed model was compared with five other machine learning methods, showing improved performance. The proposed model’s performance was verified through in-depth analysis and comparisons. The comparisons involved multiple key indicators, and experimental results demonstrate the model’s advantages across different scenarios and datasets.

The remainder of this paper is organized as follows: Section 2 introduces work by other scholars that is related to this study. Section 3 describes how the MSACNN-RM model is implemented. The experimental data, data preprocessing methods, experimental results, and analysis are described in Section 4. The conclusions are presented in Section 5.

## 2 Related work

In deep learning, effective feature extraction is crucial for recognizing sEMG data. A suitable network architecture can better capture and represent useful information in sEMG signals, thereby improving recognition performance. Therefore, the design and selection of an appropriate network structure are paramount for successful sEMG recognition. Elahe (Rahimian et al., 2021) proposed the Few-Shot learning-Hand Gesture Recognition (FS-HGR) architecture for gesture recognition, achieving classification accuracies of 85.94% and 73.6% on the Ninapro DB2 and Ninapro DB5 datasets, respectively. Chen (Chen et al., 2020) applied a transfer learning strategy to a hand gesture recognition method based on a CNN and CNN + LSTM. Compared to methods without transfer learning, the accuracy improved by 10%–38% in recognizing 30 gestures, and the training time was reduced. Li (Li et al., 2021b) used a multi-stream convolutional network with a fusion attention mechanism for sEMG gesture recognition, achieving an average accuracy of 84.39% on 52 gestures obtained from 27 subjects in the Ninapro DB1 dataset. Wang (Wang et al., 2023) proposed a parallel structure network (IRDC-net) using an architecture with residual modules and expanded convolutions to enlarge the model’s receptive field, achieving a classification accuracy of 89.82% on the Ninapro DB1 dataset. Despite the remarkable achievements in sEMG-based gesture recognition, the current accuracy of multi-gesture sEMG recognition still requires further improvement (Yu et al., 2020; Li et al., 2022).

Multi-stream CNNs have made significant strides in the field of sEMG gesture recognition. These networks can receive outputs from multiple channels, with each channel having a dedicated feature-extraction network, typically culminating in feature fusion in the final layer. This design enhances the feature extraction capabilities (Pan et al., 2022). Wang (Wang et al., 2022b) proposed a multi-stream convolutional block attention module-gated recurrent unit (MCA-GRU) network by integrating attention mechanisms, gated recurrent units, and multi-stream CNNs. They fused acceleration signals and sEMG signals, achieving an accuracy of 89.7% on the Ninapro DB1 dataset. Gu (Gu et al., 2022) employed a multi-stream CNN to extract features from different sub-images of sEMG. The features were then fused, resulting in accuracies of 92.76% on the Ninapro DB1 dataset. This outperformed traditional machine learning methods for gesture recognition. Peng (Peng et al., 2022) combined attention mechanisms, residual blocks, and multi-stream CNNs to extract multidimensional spatial features from signal morphology, electrode space, and feature map space. They fused the learned multiview depth features using a view aggregation network composed of early and late fusion networks, achieving higher gesture recognition accuracy for each participant on the Ninapro DB2 and Ninapro DB4 datasets compared with previous models.

Although existing research in the field of sEMG-based gesture recognition has yielded notable achievements, the inherent non-linearity of sEMG signals, individual variability, and susceptibility to environmental interference pose challenges. Consequently, there remains significant potential for improvement in algorithms for gesture recognition using sEMG.

## 3 Algorithm implementation

### 3.1 Multiple-stream CNN

The MSCNN architecture can handle multiple modalities. Its uniqueness lies in its ability to utilize simultaneously multiple input streams (or channels) to process different types of information (Pan et al., 2022). The multimodal processing capability of the MSCNN endows it with potential value across various application domains (Eslami and Yun, 2021; Peng et al., 2021; Niu et al., 2021; Yang et al., 2022).

The MSCNN design allows the network to accommodate multiple input streams, each capable of containing different types of data such as images, text, and other features. These input streams can undergo parallel convolution and pooling for feature extraction. Furthermore, the MSCNN adopts a multibranch structure, with each branch corresponding to an input stream. Each branch comprises independent convolution, pooling, and fully connected layers to learn specific feature types. The outputs of these branches are typically merged into the subsequent layers of the network to form the overall network output.

To leverage to the fullest extent the features learned from each input stream, the MSCNN often introduces a feature fusion layer at the last level in the network. This step, achieved through concatenation or weighted summation, helps integrate the information learned from various branches, thereby enhancing the network’s performance.

**FIGURE 1:**
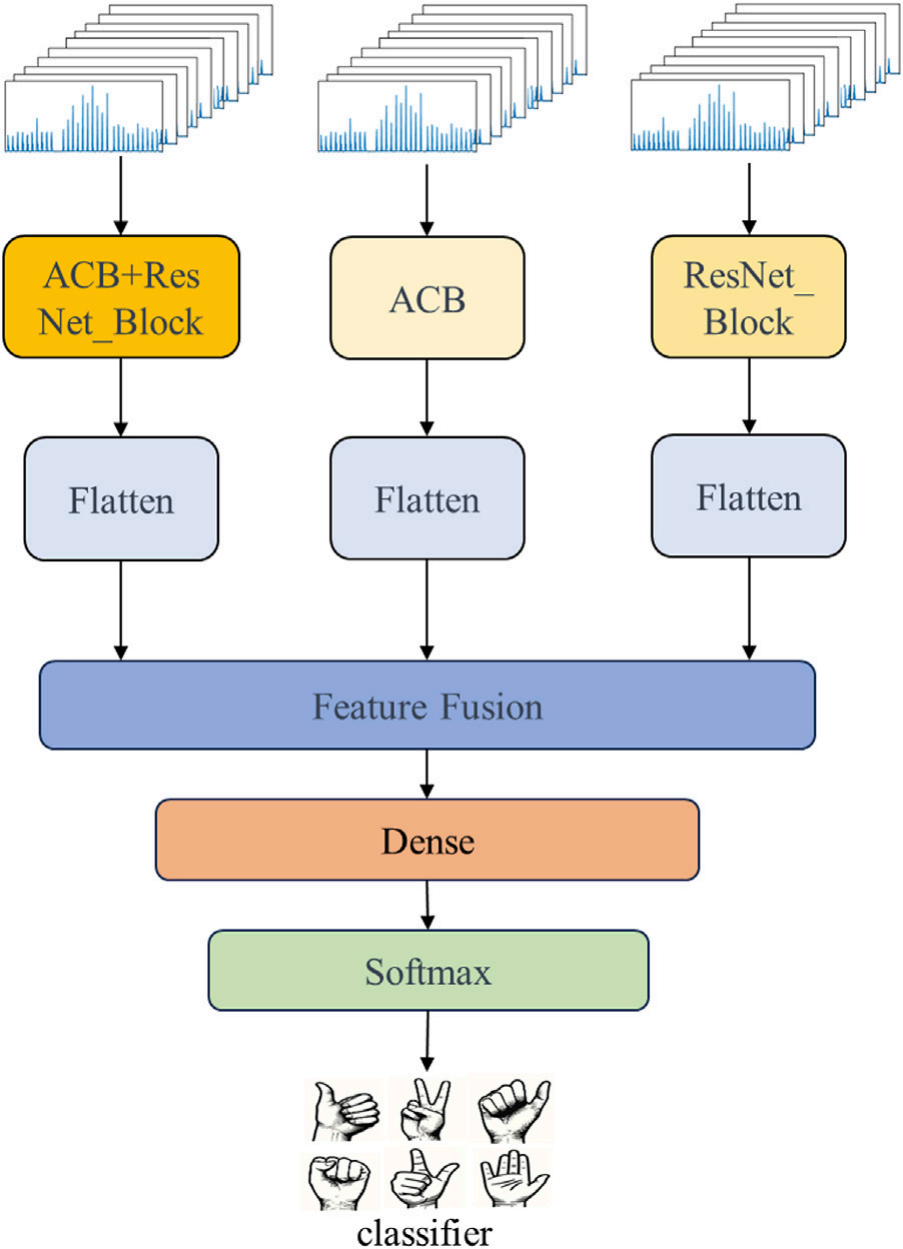
Simplified diagram of the MSCNN.

In this study, we leveraged the multi-input stream capability of the MSCNN by applying different network structures to extract features from each input stream for processing the sEMG signals. Lastly, a feature fusion operation outputs the fully connected layers as the final output. A simplified diagram of the multi-stream neural network structure used in this study is shown in the Figure 1.

### 3.2 Adaptive CNN

An adaptive CNN (ACNN) is an innovative neural network architecture (Wang et al., 2022d) primarily distinguished by introducing an adaptive mechanism. This mechanism enables the shape and weights of the convolutional kernels to be dynamically adjusted during convolution operations. The design objective was to enhance the adaptability of the model, making it more flexible and capable of effectively accommodating the spatial structures of various input data.

An ACNN dynamically adjusts input data features by replacing fixed-size convolutional kernels in traditional convolutional layers with adaptive convolutional kernels (Sang and Ruan, 2021). In contrast to traditional convolutional kernels, adaptive convolutional kernels can flexibly adjust their shapes and weights based on the features of the input data. This adaptability enables the network to capture the local features of the input data more accurately, thereby significantly improving the effectiveness of feature extraction. Additionally, ACNN demonstrates spatial adaptability, not only in terms of convolutional kernel shape and weights but also in flexible handling of the spatial structure of input data. This spatial adaptability makes the network more suitable for processing the features of different scales and shapes, significantly enhancing its capability for modeling complex input data.

To achieve this adaptability in deep learning, the learning of convolutional kernel weights and shapes is typically accomplished automatically through backpropagation, without the need for additional parameters.

In adaptive convolutional layers, the weights of the convolutional kernels are adjusted through an attention mechanism and then used to convolve the input feature maps. Below is the iterative formula description of this process: Global Average Pooling (GAP) is as shown in Equation 1:

$$GAP\left(X\right)=\frac{1}{H\times W}\overset{H}{\underset{i=1}{\sum }}\overset{W}{\underset{j=1}{\sum }}X_{ij}$$ (1)

Where X is the input feature map, and H and W are the height and width of the feature map, respectively.

Generation of Attention Weights is formulated as in Equation 2:

$$A=softmax\left(dense\left(GAP\left(X\right)\right)\right)$$ (2)

A represents the attention weights generated by a fully connected layer followed by a softmax activation function, corresponding in dimension to the number of output channels of the convolution layer (i.e., the number of filters).

Expansion of Attention Weights is given in Equation 3:

$$A^{\prime }=expand\left(A\right)$$ (3)

In this step, the attention weights A are expanded to the same four-dimensional shape as the convolutional kernels K to enable element-wise multiplication with each kernel.

Adjusted Convolutional Kernel Weights is formulated as in Equation 4:

$$K^{\prime }=K\cdot A^{\prime }$$ (4)

Where K is the original convolutional kernel, and $K^{\prime }$ is the kernel after applying the attention weights.

Convolution Operation is given in Equation 5:

$$Y=conv2d\left(X,K^{\prime }\right)$$ (5)

The adjusted kernels $K^{\prime }$ are used to perform standard convolution operations on the input feature map X .

The ACNN demonstrates the potential for widespread applications, especially when dealing with complex images, videos, or other types of spatial data. Its advantage lies in its ability to better adapt to spatial structural changes in different scenarios (Zhou et al., 2023). Therefore, when there are variations between subjects in sEMG, incorporating an ACNN can enhance the extraction of features in the signal that are unrelated to the subjects.

### 3.3 Residual network

ResNets are deep learning architectures designed to address issues such as gradients vanishing or exploding when deep neural networks are trained. Their key innovation is the introduction of residual blocks to construct deep networks, that facilitate the network’s ability to learn identity mapping (He et al., 2016).

The core of the ResNet is a residual block that includes skip or shortcut connections. These connections allow the input signal to bypass one or more layers, making it easier for the network to learn the identity mappings. For this, let the input be X and output be Y . The mathematical expression for a residual block can be represented as in Equation 6:

$$Y=X+F\left(X\right)$$ (6)

where F(X) is the output of nonlinear transformation within the residual block. The key concept introduced by ResNet is to learn residuals, by which it aims to reduce the identity mapping residuals toward zero. This approach effectively mitigates the risk of gradient vanishing because, even with an increase in network depth, learning the identity mapping residuals remains relatively straightforward. In addition, ResNet incorporates skip connections between layers, allowing the input signal to pass to subsequent layers. Through additional operations, the input signal is merged with the features learned by the subsequent layers. This connection method helps alleviate the issue of gradient vanishing, enabling the network to learn feature representations more effectively.

ResNet constructs deep networks by stacking multiple residual blocks, enabling the network to maintain a relatively good training performance, even when it is very deep. This is highly beneficial for deep learning tasks such as image classification and object detection. To reduce the computational complexity of the model, ResNet introduces the “Bottleneck” architecture, which involves arranging 1 × 1, 3 × 3, and 1 × 1 convolutional layers sequentially within a residual block. This design reduces the number of channels in the intermediate convolutional layers, thereby enhancing computational efficiency. Finally, ResNet employs global average pooling to transform the final feature map into a vector that can be used for classification tasks.

The introduction of ResNet has propelled the development of deep learning significantly, making it feasible to design deeper neural networks. Its improved performance in large-scale image recognition competitions, such as the ImageNet Challenge, demonstrates its effectiveness in practical applications. The principles of ResNet have also been widely applied in the designs of other deep learning architectures (Alaeddine and Jihene, 2021; Shen et al., 2021; Dutande et al., 2022), providing crucial insights for enhancing the training and performance of models.

The residual block receives an input feature map. The initial convolutional layer processes the input feature map with a kernel size of (2,1). The stride of the convolutional layer is set to 1, and “SAME” padding is used to ensure the size of the output feature map remains unchanged. The output of this convolutional layer is then passed through a batch normalization (BN) layer to stabilize the training process and accelerate convergence. Subsequently, the result is transformed non-linearly using the ReLU activation function. The mathematical expression for the ReLU function is as follows in Equation 7:

$$f\left(x\right)=max\left(0,x\right)$$ (7)

**FIGURE 2:**
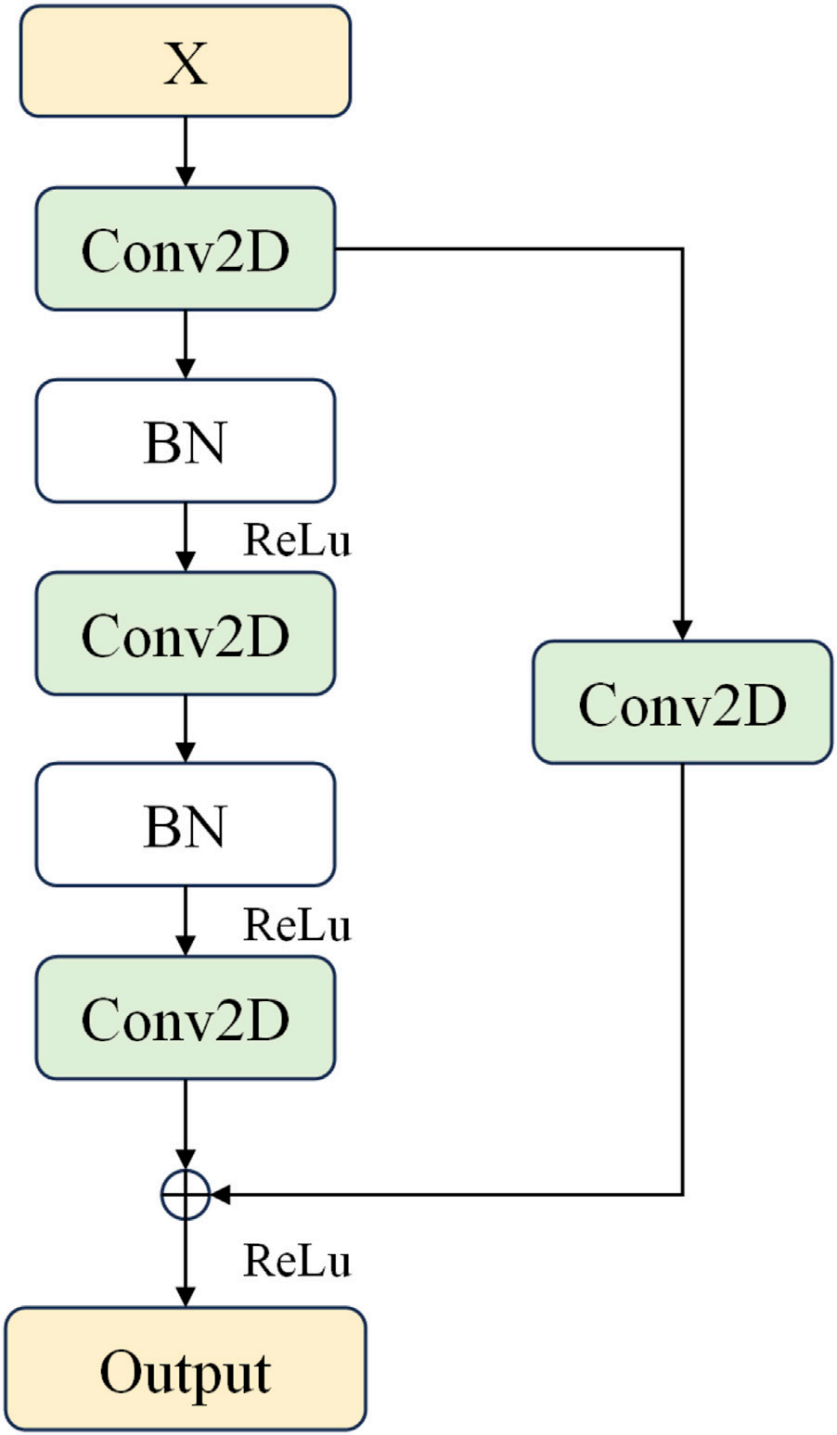
Residual block used in this study.

This implies that if the input value is positive, then the output is the value itself; if the input value is negative, the output is zero. Utilizing the ReLU function can enhance computational efficiency and the generalization capability of the network, and it can also address the issue of vanishing gradients during the training of deep networks. A secondary convolutional layer applies the same configuration (i.e., same kernel size, stride, and padding) to the output of the initial convolutional layer. Similarly, the output from this layer is also subjected to BN and processed through the ReLU activation function. A residual connection allows the original input to be processed by a convolutional layer with a kernel size of (1,1) and a stride of 1 (to match dimensions and adjust stride), ensuring it can be added to the output of the secondary convolutional layer. This transformed inputis then added to the output of the secondary convolutional layer to form a residual connection. This operation facilitates the flow of information and prevents gradient vanishing issues in deep networks. Finally, the resultant feature map after addition is processed through the ReLU activation function to produce the final output feature map. In this article, the residual blocks used are depicted in Figure 2.

### 3.4 Multi-stream adaptive CNN with residual modules

This study introduced a multi-stream adaptive CNN with residual modules. A multi-stream neural network has three inputs and each identical input passes through a different network architecture. These architectures include multiple adaptive convolutional layers, residual blocks, MaxPooling layers, activation layers, feature-fusion layers, convolutional layers, flattened layers, and fully connected layers. The residual blocks used in this context differed only in the number of convolutional kernels.

**FIGURE 3:**
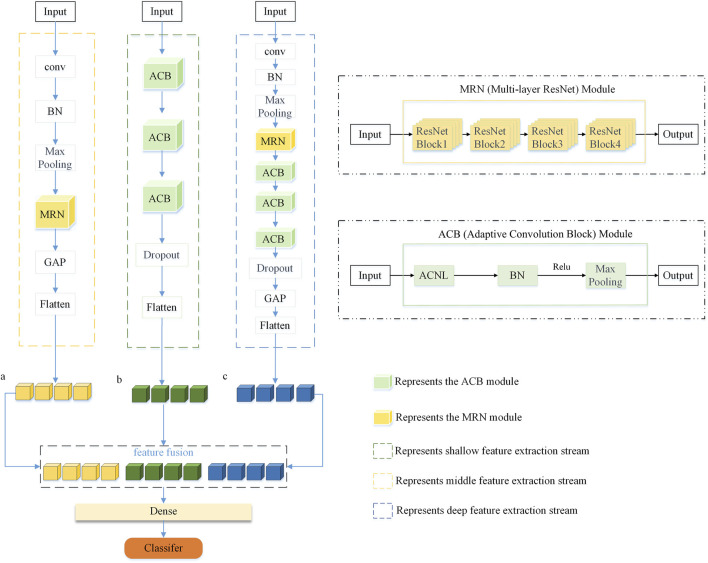
The figure depicts the MSACNN-RM process for sEMG signal analysis. Features extracted via sliding window technique are fed into the model, producing three feature vectors, a, b, and c. These vectors are then fused and processed through a fully connected layer, followed by the application of a softmax function for feature classification.

The first convolutional stream is composed as follows: The data initially undergo a convolutional layer with 64 convolutional kernels and a kernel size of (2, 1). The ReLU activation function, zero-padding, BN, and MaxPooling layer are employed to prevent data loss and stabilize the neural network learning process. Subsequently, ResNet residual blocks with 64, 128, 256, and 512 convolutional kernels are connected. These four residual blocks are defined as the MRN module in Figure 3. Subsequently, three layers of adaptive convolutional neural layer (ACNL), each initialized with 32 convolutional kernels and a kernel size of 1, are employed. After each ACNL, connections to a BN layer, a ReLU activation layer, and a MaxPooling layer 2 × 2 in size, are made. In Figure 3, we define the module composed of ACNL, BN, ReLU, and MaxPooling as the ACB module. Finally, the output is obtained through a dropout layer with a dropout rate of 50%, a global average pooling layer, and a flattened layer. The design goal of the first convolutional stream is adaptively to extract deep features from the sEMG signals.

The second convolutional stream functions as follows: The sEMG feature maps are fed into three layers of adaptive convolution for feature extraction, with each layer initially equipped with 32 kernels, each of size (1,1). After each layer of adaptive convolution, there are connections to a BN layer, a ReLU activation layer, and a MaxPooling layer 2 × 2 in size. This design can help stabilize the model during training, accelerate the convergence rate, reduce the number of parameters and computational load, and enhance the model’s robustness and adaptability to changes in input. Finally, the output is obtained through a dropout layer with a dropout rate of 50% and a flattened layer. The second convolutional stream is included to enhance the model’s adaptive ability to extract features from sEMG signals.

The third convolutional stream functions as follows. The input data first enter a convolutional layer with 64 convolutional kernels and a kernel size of (2, 1). The ReLU activation function is applied, and zero-padding is used. After this convolutional layer, the network sequentially connects a BN layer and a MaxPooling layer. The BN layer standardizes the distribution of input batches to mitigate gradient anomalies, while the MaxPooling layer adopts a 2 × 2 window (stride = 2) to preserve locally salient features and reduce spatial dimensions. The ResNet residual blocks are connected with 64, 128, 256, and 512 convolutional kernels. Finally, the output is obtained through a global average pooling layer and a flattened layer. The third convolutional stream is used to extract deep features from the sEMG signals.

Finally, the outputs from all the convolutional streams converge to the fusion layer and are fused column-wise. The fused data then enter the output layer with 52 neurons, which utilizes softmax as an activation function to transform the output into a probability distribution. The final model architecture is illustrated in Figure 3.

## 4 Experimentation

### 4.1 Dataset

The Ninapro dataset which has been widely used in sEMG research (Li et al., 2023b; Soroushmojdehi et al., 2022; Xiong et al., 2023) was used for the testing and training. It consists of ten complete data subsets, labeled DB [1–10], covering sEMG signal files both from amputees and non-amputees. To ensure the generalization and effectiveness of the proposed model across different datasets, we selected DB1, DB2, and DB4 for training and testing. The diversity of gestures in the Ninapro dataset makes it widely applicable for research on gesture recognition. Additional data can further evaluate the model’s ability to extract essential features from sEMG signals.

In DB1, ten electrodes (MyoBock 13E200, Ottobock SE & Co. KGaA) were used to record the sEMG signals (Atzori et al., 2014). Among them, eight were evenly distributed on the forearm at a height corresponding to the radius-elbow joint, whereas the remaining two electrodes captured signals from primary activity points on the flexor and extensor muscles. The dataset comprised the sEMG data from 27 healthy subjects who performed 52 hand gestures. These included basic finger movements, isometric and isotonic hand gestures, fundamental wrist movements, grasping, and functional motions.

DB2 utilized 12 active double-differential wireless electrodes from a wireless sEMG system (Delsys Trigno, Delsys Inc.) and sampled the sEMG signals at 2,000 Hz (Atzori et al., 2014). Eight electrodes were evenly distributed on the forearm corresponding to the radius of the elbow joint. Two electrodes were placed at the main activity points of the flexor and extensor muscles and two additional electrodes were positioned at the main activity points of the biceps and triceps brachii muscles. The dataset included 49 gesture actions repeated 10 times, involving basic finger movements, grasping, and functional motions. The participants were instructed to repeat the actions with their right hand. Each repetition lasted for 5 s, followed by a 3-s rest to prevent muscle fatigue. The experimental design involved 49 different actions (with rest intervals) performed by 40 participants.

In DB4, the sEMG data were collected using 12 electrodes (Cometa Systems Inc.) at a sampling rate of 2000 Hz (Pizzolato et al., 2017). Eight electrodes were evenly distributed on the forearm, corresponding to the height of the radius-elbow joint. Two captured sEMG signals from the flexor and extensor muscles, whereas the last two recorded signals from the biceps and triceps brachii muscles. The dataset included 52 hand movements repeated six times, including isometric and isotonic hand configurations, basic wrist movements, fundamental finger actions, grasping, and functional motions. The data covered sEMG recordings from 10 subjects who repeated 52 hand movements, including static postures. Each repetition of an action lasted approximately 5 s, followed by a 3-s rest to prevent muscle fatigue. During data collection, the participants were instructed to use their right hand to repeat these actions.

The dataset was randomly divided into three groups: one for use as the training set, the second as the test set, and the last to generate a confusion matrix. The training set accounted for 70% of the total dataset, the test set for 15%, and the validation set for 15%. The experimental hardware environment comprised an Intel(R) Core(TM) i9-13900K CPU @ 5.8 GHz with 32 GB of memory, and all experiments were implemented using TensorFlow 2.10.0 + cu160 on an NVIDIA RTX 4090 GPU.

### 4.2 Data preprocessing

When dealing with time-series signals such as sEMG, the sliding window method effectively captures the temporal features of muscle electrical signals, thereby improving the performance of tasks such as gesture recognition (Bu et al., 2023). Therefore, in this study, the data were segmented using a sliding-window approach. Considering the acceptable delay range for the human body (Côté-Allard et al., 2019), the window size was set to 256 ms, and the sliding step was set to 100 ms.

Before training the model, a series of preprocessing steps were performed. Firstly, the data were randomly shuffled to avoid overfitting and to increase the diversity of the training samples, effectively mitigating the potential impact of the data’s native ordering. This approach significantly benefits support for mini-batch stochastic gradient descent and enhances training efficiency.

Secondly, the data underwent one-hot encoding, transforming the labels into a one-hot encoded form. This step is crucial for handling multiclass problems, adapting to neural networks and deep learning models, improving model performance, mitigating the impact of training with numerical labels, and supporting multi-output scenarios.

**FIGURE 4:**
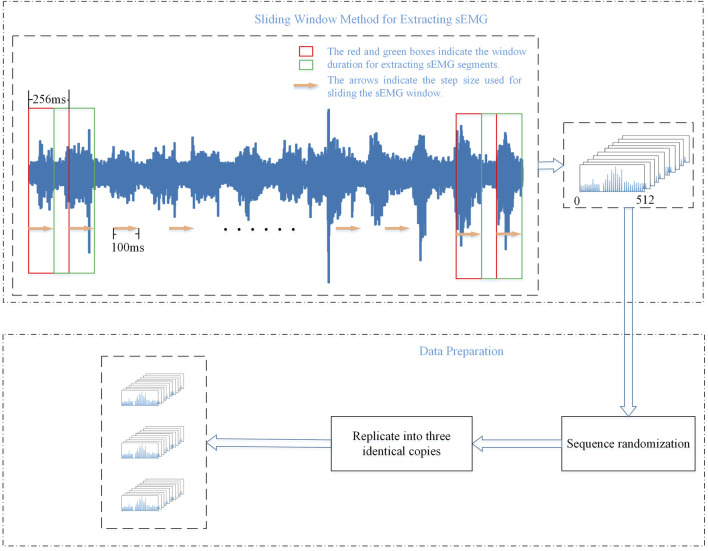
Flow chart of sliding window method and the preparation of training data.

Finally, the training and test sets were split into three parts to accommodate multiple streams of the CNNs. The aforementioned preprocessing steps ensure the robustness and efficiency of model training, allowing it to better adapt to the complexity of gesture recognition tasks. These steps help the model better adapt to the features of different gestures when learning sEMG, ultimately improving the accuracy and robustness of gesture recognition tasks. This approach aims to utilize the information in the data more reasonably and improve its suitability for training deep-learning models. Figure 4 presents the processing pipeline for sEMG signals in this article.

### 4.3 Experimental results and analysis

The model employed the Adam optimizer with categorical cross-entropy as the loss function and accuracy as the performance metric for both training and evaluation. The training consisted of 150 epochs with a batch size of 32. Ultimately, on the Ninapro DB1 dataset, the model was trained on data from subjects 1–5, 7, 9, and 11–13, achieving a training set accuracy of 99.86% and a maximum testing set accuracy of 98.24%. On the Ninapro DB2 dataset, the model was trained on data from subjects 1-3 and 5-8, resulting in a training set accuracy of 99.78% and a maximum testing set accuracy of 93.52%. Similarly, on the Ninapro DB4 dataset, the training involved data from subjects 1–4, 6, and 8–10, producing a training set accuracy of 99.85% and a maximum testing set accuracy of 92.27%. These results validate the effectiveness of the proposed model. The Table 1 displays the final training results.

**TABLE 1:** Training and test set accuracy of MSACNN-RM across datasets DB1, DB2, and DB4.

Dataset name	Training set Accuracy (%)	Test set Accuracy (%)
DB1	99.86	98.24
DB2	99.78	93.52
DB4	99.85	92.27

#### 4.3.1 Evaluation of model performance using K-fold cross-validation

To better demonstrate the experimental results, we conducted a K-fold cross-validation analysis to provide more reliable outcomes. K-fold cross-validation is a widely used validation method that involves dividing the dataset into K subsets and performing K iterations of training and testing. In each iteration, a different subset is used as the test set, while the remaining subsets are used as the training set. After completing all K iterations, the average of all test results is calculated as the final performance evaluation metric for the model. This process helps to reduce the bias associated with a single partition, providing a more reliable model evaluation. This method effectively reduces bias and variance in model evaluation, thereby enhancing the reliability and robustness of the results. In this experiment, we set the value of K to 5 and introduced Precision, Recall, and F1 Score as additional evaluation metrics. The final results are shown in Table 2. From Table 2, it can be concluded that the model’s performance on dataset DB1 is significantly superior to the other two datasets, with an average accuracy and precision close to 97%. In terms of standard deviation, the model on dataset DB1 is the most stable (with a standard deviation ranging from 0.0049 to 0.0052). Overall performance metrics indicate that dataset DB1 excels in all aspects, making it suitable for high-precision applications. The performance of datasets DB2 and DB4 is comparable; however, DB4 exhibits greater variability, indicating a need for further optimization. In summary, the model achieves satisfactory performance and stability across all three datasets.

**TABLE 2:** Performance metrics of MSACNN-RM after K-fold cross-validation on datasets DB1, DB2, and DB4.

Dataset	Average accuracy	Recall	F1	Precision	Std
DB1	0.9685	0.9685	0.9693	0.9701	0.0049–0.0052
DB2	0.9059	0.9059	0.9124	0.9189	0.0091–0.0095
DB4	0.9053	0.9053	0.9102	0.9152	0.0182–0.0194

#### 4.3.2 Loss analysis and confusion matrix analysis

**FIGURE 5:**
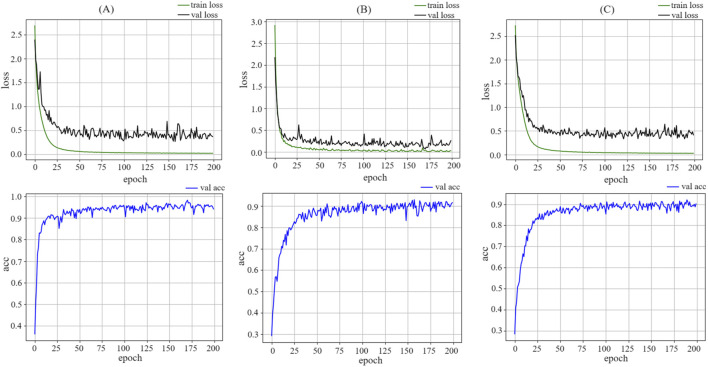
The figure illustrated the changes in loss and accuracy curves for the training and testing sets during the model training process. Area **(a)** shows the training process of MSACNN-RM on DB1, area **(b)** displays the training on DB2, and area **(c)** represents the training on DB4. The image clearly demonstrates that the model proposed in this paper possesses robust data fitting capabilities.

The model training on the DB1, DB2 and DB4 dataset is illustrated in Figure 5. On the DB1,The loss curve exhibited rapid convergence, with learning gradually slowing after approximately 20 epochs and eventually stabilizing. On the DB2 and DB4 datasets, the loss curve also demonstrates rapid convergence, with learning gradually slowing after approximately 40 epochs and eventually stabilizing. This indicates that the model rapidly learns the general features of the data in the early stages and achieves better fitting results through deeper learning in later stages. As the training progressed, the model adjusted the parameters to better fit the training data. The confusion matrix reveals that the model performs well for each category. The predicted results for each category matched the actual labels, indicating that the model was capable of capturing the features of different categories. The training dynamics and performance demonstrated the effectiveness and robustness of the model. At approximately 100 epochs, the model approached a balanced state, and the learned features fit the training data well. The final results indicate that the model achieved its optimal accuracy at 171 epochs on DB1, at 158 epochs on DB2, and at 187 epochs on DB4. This was a satisfactory outcome, indicating that the model performed convincingly on the DB1, DB2, and DB4 datasets.

**FIGURE 6:**
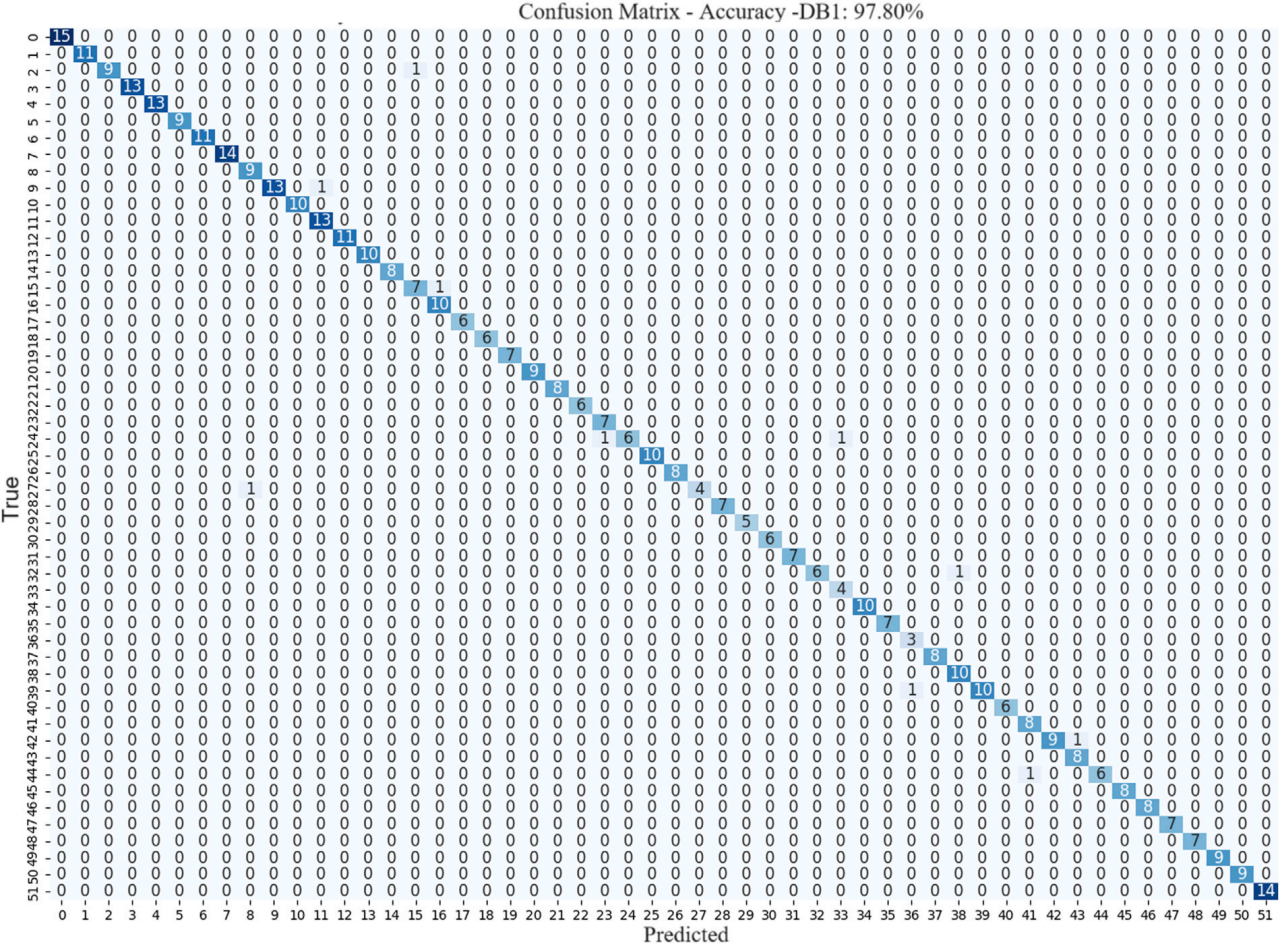
Confusion Matrix of MSACNN-RM on DB1 validation dataset.

The confusion matrix provides crucial insights into the performance of the model for different gesture classification tasks. The confusion matrices for DB1 in Figure 6 respectively confirm that the model exhibited excellent performance in gesture recognition tasks, achieving high accuracy for most gestures. However, there was a small proportion of misclassifications in the confusion matrices. These errors can be attributed to the similarity between the sEMG signals of certain gestures, making it difficult for the model to differentiate them accurately.

#### 4.3.3 Ablation experiments

**FIGURE 7:**
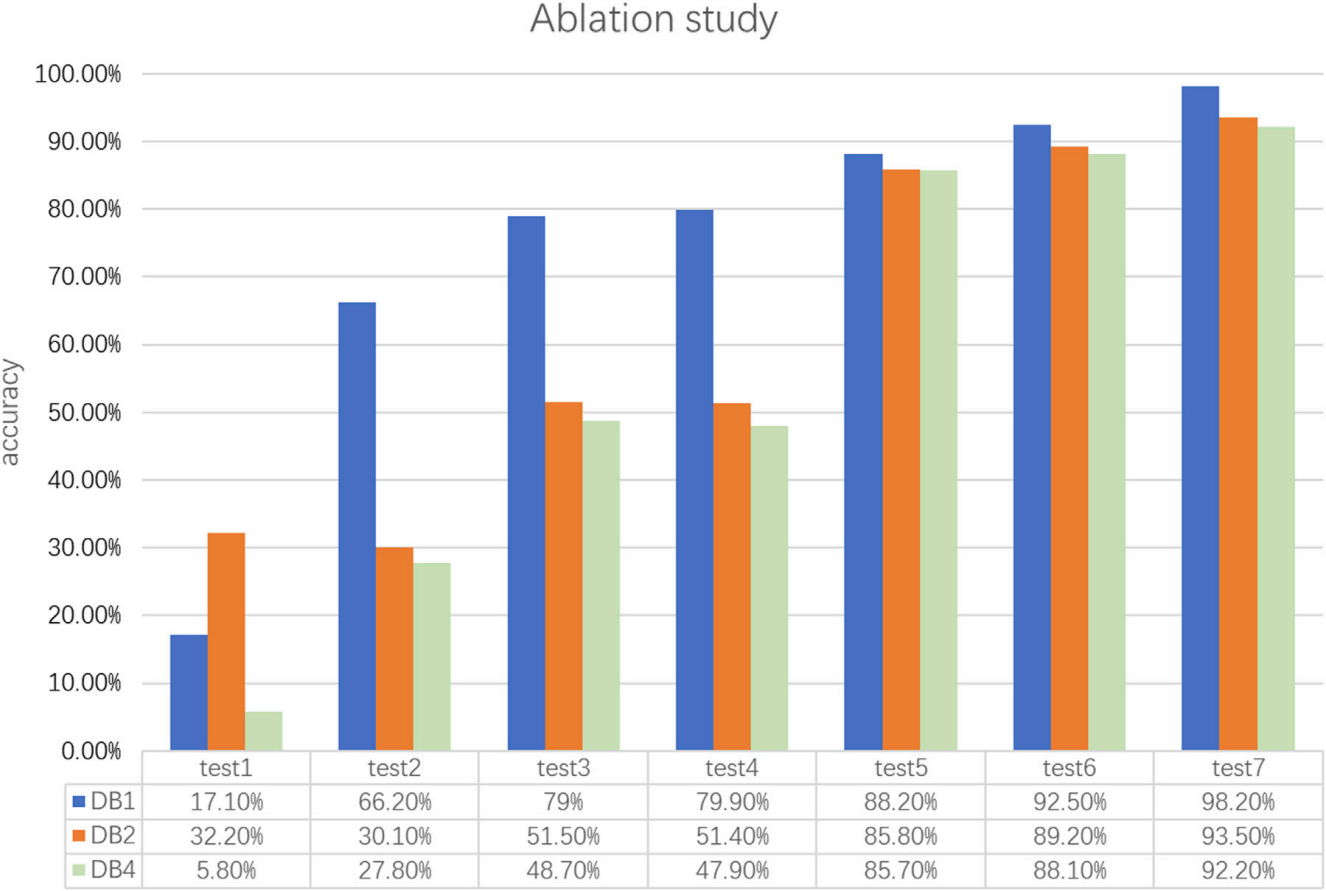
The figure shows the ablation experiment results of different models on the DB1, DB2, and DB4 datasets. MSACNN-RM demonstrates statistically significant differences (P < 0.05) compared to the other models in accuracy.

To further validate the effectiveness of the MSACNN-RM in sEMG gesture recognition, we conducted ablation experiments by fine-tuning the adjustments to the network structure as shown in Table 3. A standardized multi-stream CNN (MSACNN) was used as the baseline model for five experiments. The sliding window method was employed to process the sEMG images as model inputs. Table 3 lists the different model configurations that were tested. Figure 7 illustrates the accuracies of the different model configurations for DB1, DB2, and DB4.

**TABLE 3:** Overview of network structures evaluated in experimental tests (Test1 to Test7).

Test	Network structure
Test1	MSCNN
Test2	MSCNN + BN
Test3	MSCNN + BN + MaxPooling
Test4	MSCNN + BN + MaxPooling + ACNN
Test5	MSCNN + BN + MaxPooling + ResNet
Test6	MSCNN + BN + MaxPooling + ResNet + ACNN
Test7	MSCNN + BN + MaxPooling + ResNet + Integration of ACNN Module and ResNet

To comprehensively evaluate the effectiveness of MSACNN-RM in sEMG-based gesture recognition, we compared it with four other models: Test4, Test5, Test6, and Test7. All models were tested using a 5-fold cross-validation method on the same datasets (DB1, DB2, and DB4). The evaluation metric used was Accuracy. A Shapiro-Wilk test was conducted to verify the normality of the results, and all outcomes conformed to the normal distribution assumption. Consequently, paired t-tests were employed to assess the performance differences between models. The test results indicated that MSACNN-RM exhibited statistically significant improvements over the comparison models in Accuracy (P < 0.05).

By comparing the training results of different models on the same dataset, the significant impact of BN on recognition accuracy can be clearly observed. The unique advantage of BN lies in reducing the internal covariate shift, which alleviates the model’s sensitivity to parameter initialization and choice of learning rate. This effectively optimizes the gradient descent and accelerates the convergence. Therefore, introducing BN helps fit the dataset better and achieves a higher recognition accuracy.

Comparing the DB1, DB2, and DB4 training results in Figure 7, the introduction of adaptive convolution and ResNet into the multi-stream convolutional network significantly improved the model’s recognition accuracy. This advantage was most pronounced for DB1. The inclusion of adaptive convolution layers increased the average recognition accuracy of 52 gestures to 79.9%, and ResNet further increased the average recognition accuracy to 88.2%. By combining the ACNN and ResNet, the MSACNN-RM achieved an average recognition accuracy of 98.24% for DB1. This combination maximizes the advantages of both and significantly advances feature learning and extraction. The gesture recognition accuracy of MSACNN-RM also shows significant improvements on DB2 and DB4.

#### 4.3.4 Comparison with other network models

The exceptional performance of the proposed MSACNN-RM network model was validated by a detailed comparison with other recent research models, particularly on the Ninapro DB1 dataset. This dataset was aligned with the dataset employed in this study and comprised 52 different hand gestures by 27 healthy subjects, enabling a direct and intuitive comparison of the experimental results. A horizontal comparison confirmed that the MSACNN-RM network algorithm outperformed the other algorithms regarding recognition accuracy, as shown in Table 4.

**TABLE 4:** Comparison of gesture recognition models and their accuracy on the DB1 dataset.

Name	Model structure	Accuracy (%)
Zheng (Zheng et al., 2022)	Adaptive K Nearest Neighbor,KNN	68.04
He (He et al., 2018)	LSTM + MLP	75.45
Geng (Geng et al., 2016)	ConvNet	77.80
Hu (Hu et al., 2018)	CNN-RNN	87.00
Yang (Yang et al., 2021)	MResLSTM	89.65
Xu (Xu et al., 2022)	CFF-RCNN	88.87
Wang (Wang et al., 2023)	IRDC-net	89.82
Gu (Gu et al., 2022)	multi-stream CNN	92.76
ours	MSACNN-RM	98.24

Based on Table 4, it is evident that there are variations in structural design and performance among the different models. Zheng (Zheng et al., 2022) employs the Adaptive K Nearest Neighbor (Adaptive KNN) method, which, although simple to implement and computationally efficient, is sensitive to noisy data and highly dependent on feature extraction, resulting in lower recognition accuracy. He (He et al., 2018) combines Long Short-Term Memory networks (LSTM) with Multi-Layer Perceptron (MLP), effectively capturing temporal features and suitable for handling sequential data, but the model is highly complex and requires longer training times. Geng (Geng et al., 2016) utilizes convolutional neural networks for feature extraction, offering a relatively simple structure that effectively extracts spatial features, yet its ability to distinguish complex gestures is somewhat limited. Hu (Hu et al., 2018)’s CNN-RNN model integrates the strengths of both CNN and RNN, enabling the extraction of spatial features and the capture of temporal features, thereby improving recognition accuracy. However, the training process is complex and the model has a large number of parameters. Yang (Yang et al., 2021)’s MResLSTM model enhances the model’s generalization capability through multi-resolution feature extraction, achieving high accuracy but demanding significant computational resources and longer training times. Xu (Xu et al., 2022) proposes the CFF-RCNN model, which introduces multi-layer feature fusion techniques to further enhance recognition precision, though the model structure is complex. Wang (Wang et al., 2023)’s IRDC-net model possesses deep feature extraction capabilities, demonstrating strong representational power. Finally, Gu (Gu et al., 2022) employs a Multi-stream CNN structure, effectively enhancing feature extraction capability and achieving a high accuracy of 92.76%, but with a complex model structure and high computational resource requirements.

As shown in Table 4, despite significant progress made by previous studies on the Ninapro DB1 dataset, recognition accuracy has struggled to surpass 93%. The method proposed in this study achieves higher accuracy compared to the above methods. Additionally, the combination of deep learning modules used in past methods was relatively simple. In contrast, our study designs shallow, intermediate, and deep feature extraction streams, effectively enhancing the feature extraction capability for sEMG through the rational integration of ACNL and Residual modules. Moreover, the flexibility of the ACNL layer also strengthens the model’s ability to adapt to individual differences in sEMG. However, the more complex convolutional structure of the model, compared to those of previous models, incurs greater computational costs.

## 5 Conclusion

To address challenges such as inadequate effective feature extraction in sEMG-based deep learning models, poor gesture recognition speed, and low accuracy in sparse sEMG signals, this paper introduces a novel multi-stream adaptive CNNs, MSACNN-RM. By leveraging multi-stream convolution, the model explores features of sparse sEMG signals from multiple acquisition channels. The inclusion of adaptive convolution modules and ResNet blocks enhances the model’s ability to learn crucial gesture features, with a focus on more differentiated signal regions. Simultaneously, the network adaptively learns features from different feature maps, reinforcing feature extraction, ensuring accurate gesture classification, and accelerating model convergence.

The network processes electromyographic signals into model inputs using a sliding window approach, allowing the precise identification of 52 gestures. The introduction of adaptive convolution modules in multi-stream convolution effectively mitigates overfitting. Moreover, the use of residual blocks further enhances the network’s ability to extract features from sEMG signals, thus improving recognition accuracy. MSACNN-RM achieved satisfactory results on the Ninapro DB1, DB2, and DB4 datasets, demonstrating its broader potential applications in fields such as human-computer interaction and electromyographic gesture recognition.

Future research will focus on the differences in electromyographic signals caused by variations in body fat levels, disparities in arm skeletal size, and differences in hand movement capabilities among individuals. This calls for a deeper investigation into the development of a universal multi-gesture recognition algorithm. Additionally, since the model’s extensive use of convolutional computations results in a significant computational load, future research will prioritize optimizing and streamlining this network model to enhance its suitability for resource-constrained human-computer interaction platforms.

## Data Availability

The raw data supporting the conclusions of this article will be made available by the authors, without undue reservation.
